# MRI-Assisted Planning for Spinal Anesthesia in a Pregnant Woman with Radiographic Spina Bifida Occulta: A Case Report

**DOI:** 10.3390/reports9030246

**Published:** 2026-07-29

**Authors:** Misaki Inoue, Akira Motoyasu, Shogo Ema, Joho Tokumine, Kiyoshi Moriyama

**Affiliations:** Department of Anesthesiology, Kyorin University School of Medicine, 6-20-2 Sinkawa, Mitaka 181-8611, Tokyo, Japan; misaki-inoue@ks.kyorin-u.ac.jp (M.I.); motoyasu@ks.kyorin-u.ac.jp (A.M.); shogo-ema@ks.kyorin-u.ac.jp (S.E.); mokiyo@ks.kyorin-u.ac.jp (K.M.)

**Keywords:** spina bifida, spinal anesthesia, cesarean section, magnetic resonance imaging, preprocedural ultrasound

## Abstract

**Background and Clinical Significance:** Spina bifida occulta may be associated with occult spinal dysraphism, including a low-lying conus medullaris or tethered cord, which can increase the risk of neurological injury during neuraxial anesthesia. We report a case in which preoperative magnetic resonance imaging (MRI) and lumbar ultrasonography supported individualized planning for spinal anesthesia for cesarean delivery. **Case Presentation:** A 31-year-old woman at 37 weeks of gestation was scheduled for elective cesarean delivery because of marginal placenta previa. She had chronic low back pain, and previous lumbar radiography and computed tomography had demonstrated radiographic spina bifida occulta. Preoperative lumbar MRI confirmed that the conus medullaris terminated normally at L1 and showed no evidence of tethered cord, filum terminale thickening, spinal lipoma, or abnormalities at the planned L3/4 puncture site. Based on these findings and discussion with the patient, spinal anesthesia was selected. Immediately before the procedure, lumbar ultrasonography was used to identify the L3/4 interspace, visualize the posterior complex, and estimate needle depth. Spinal anesthesia was successfully achieved with a 27-gauge pencil-point needle and intrathecal hyperbaric bupivacaine, morphine, and fentanyl. Cesarean delivery was completed without new neurological deficits or major anesthetic complications. **Conclusions:** Preprocedural MRI and lumbar ultrasonography supported individualized anatomical assessment and anesthetic planning in this patient.

## 1. Introduction and Clinical Significance

The term spina bifida occulta has been used to describe a heterogeneous spectrum of abnormalities ranging from isolated non-fusion of the posterior vertebral arch to occult spinal dysraphism involving neural structures. From an anesthetic perspective, associated abnormalities such as a low-lying conus medullaris, tethered cord, and spinal lipoma are particularly important because neural tissue may extend into the conventional lumbar puncture range, increasing the risk of direct neural injury during neuraxial procedures [1,2]. Contemporary reviews therefore recommend individualized anesthetic planning based on clinical assessment and relevant spinal imaging rather than on the diagnostic label of spina bifida occulta alone [1,2].

We report a pregnant woman with radiographic spina bifida occulta in whom preoperative MRI confirmed a normally positioned conus medullaris and excluded tethered cord and other neural abnormalities. Lumbar ultrasonography was subsequently used to assess the intended puncture site, and these complementary imaging findings supported individualized planning for spinal anesthesia for cesarean delivery.

## 2. Case Presentation

A 31-year-old woman (height, 154 cm; weight, 75 kg; body mass index, 31.6 kg/m^2^) was scheduled for elective cesarean delivery at 37 weeks of gestation because of marginal placenta previa. She had gestational diabetes, gestational hypertension, and mild hyperthyroidism, and a history of asthma, attention-deficit/hyperactivity disorder, and depression. She had a soy allergy and a history of allergy to cephalosporin antibiotics. Because of a previous anaphylactic reaction to soy milk, she carried an epinephrine auto-injector.

**Figure 1:**
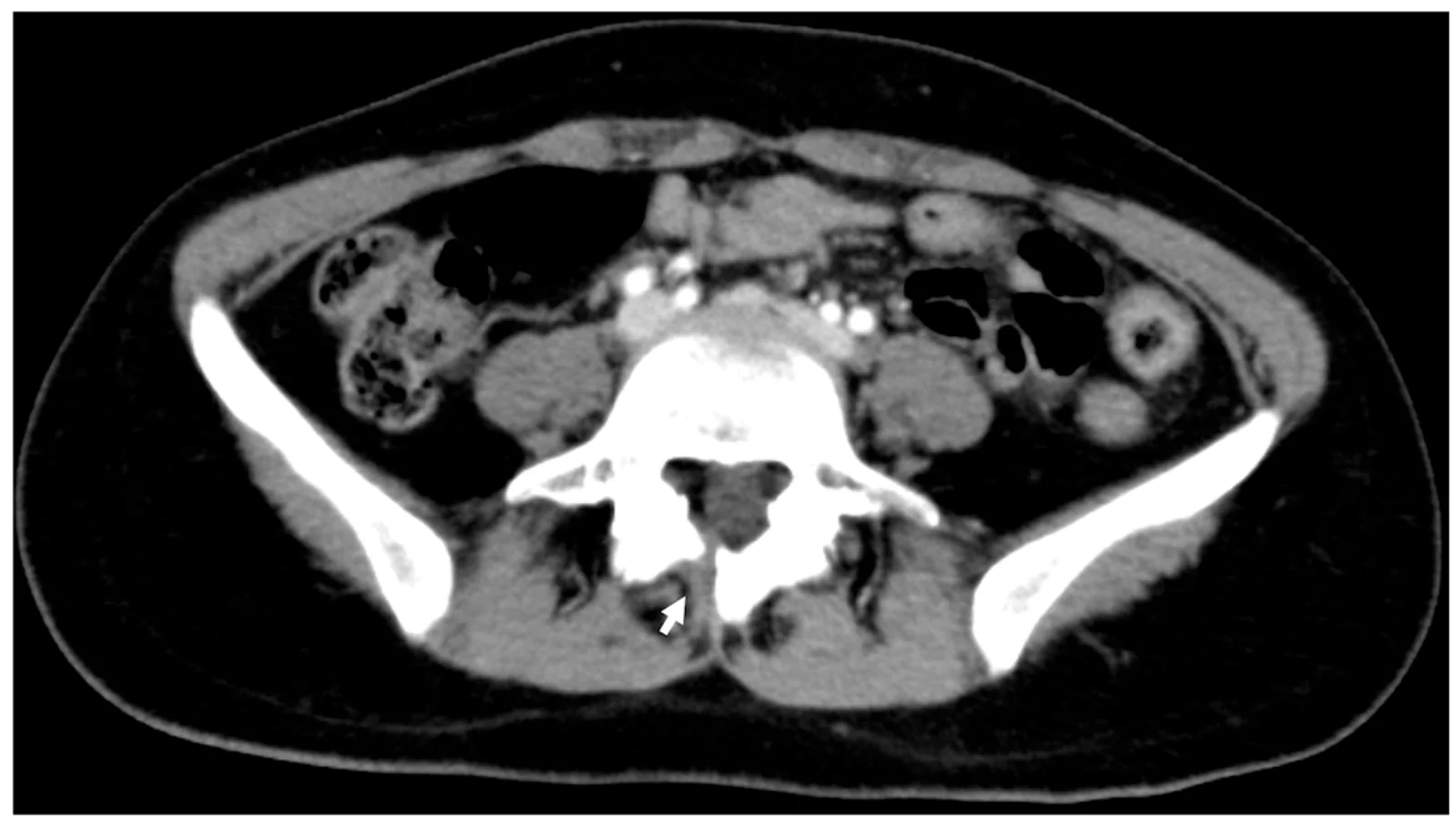
Lumbar computed tomography.

**Figure 2:**
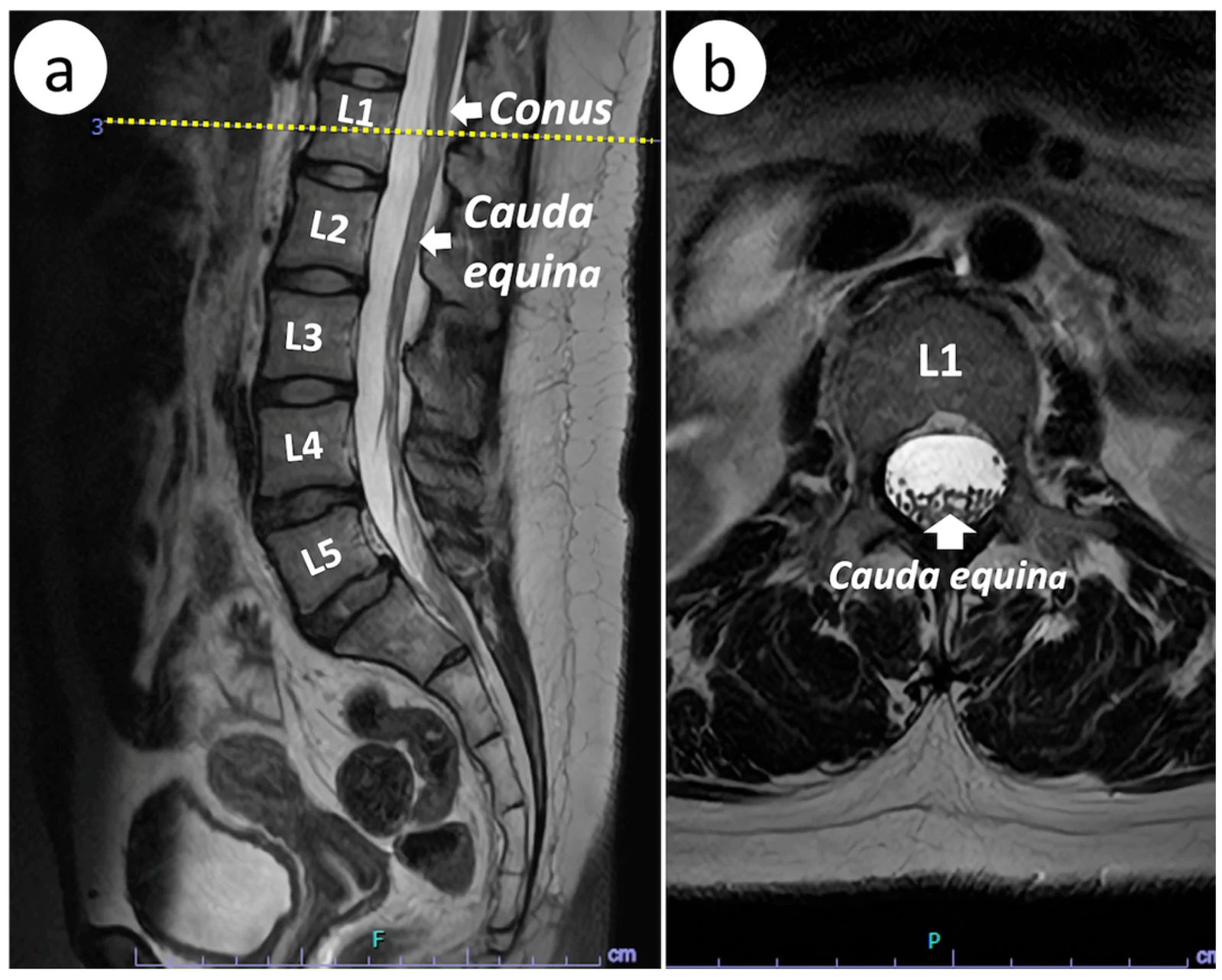
Lumbar magnetic resonance imaging. (**a**) Sagittal T2-weighted magnetic resonance imaging demonstrating a normally positioned conus medullaris (Conus) terminating at the L1 level without evidence of tethered cord. (**b**) Axial T2-weighted magnetic resonance imaging obtained at the L1 level (yellow dotted line in panel (**a**)).

At 25 years of age, lumbar radiography performed for chronic low back pain raised suspicion of L5 spondylolysis and spina bifida occulta, which was subsequently confirmed by computed tomography (Figure 1). At the preanesthetic consultation, her previous lower back pain had improved, and there were no other notable neurological symptoms. She expressed a strong preference for spinal anesthesia. She was informed that the final anesthetic plan would depend on the MRI findings and that general anesthesia would be selected if clinically relevant neural abnormalities, such as a low-lying conus medullaris, tethered cord, or spinal lipoma, were identified. Preoperative lumbar MRI demonstrated a normally positioned conus medullaris terminating at the L1 level, with no evidence of tethered cord, filum terminale thickening, spinal lipoma, or abnormalities at the planned L3/4 puncture site (Figure 2). After the MRI findings and the relative risks and benefits of neuraxial and general anesthesia were discussed with the patient and her family, spinal anesthesia was selected through shared decision-making as the first-line approach, with a plan to convert to general anesthesia if spinal anesthesia proved inadequate.

**Figure 3:**
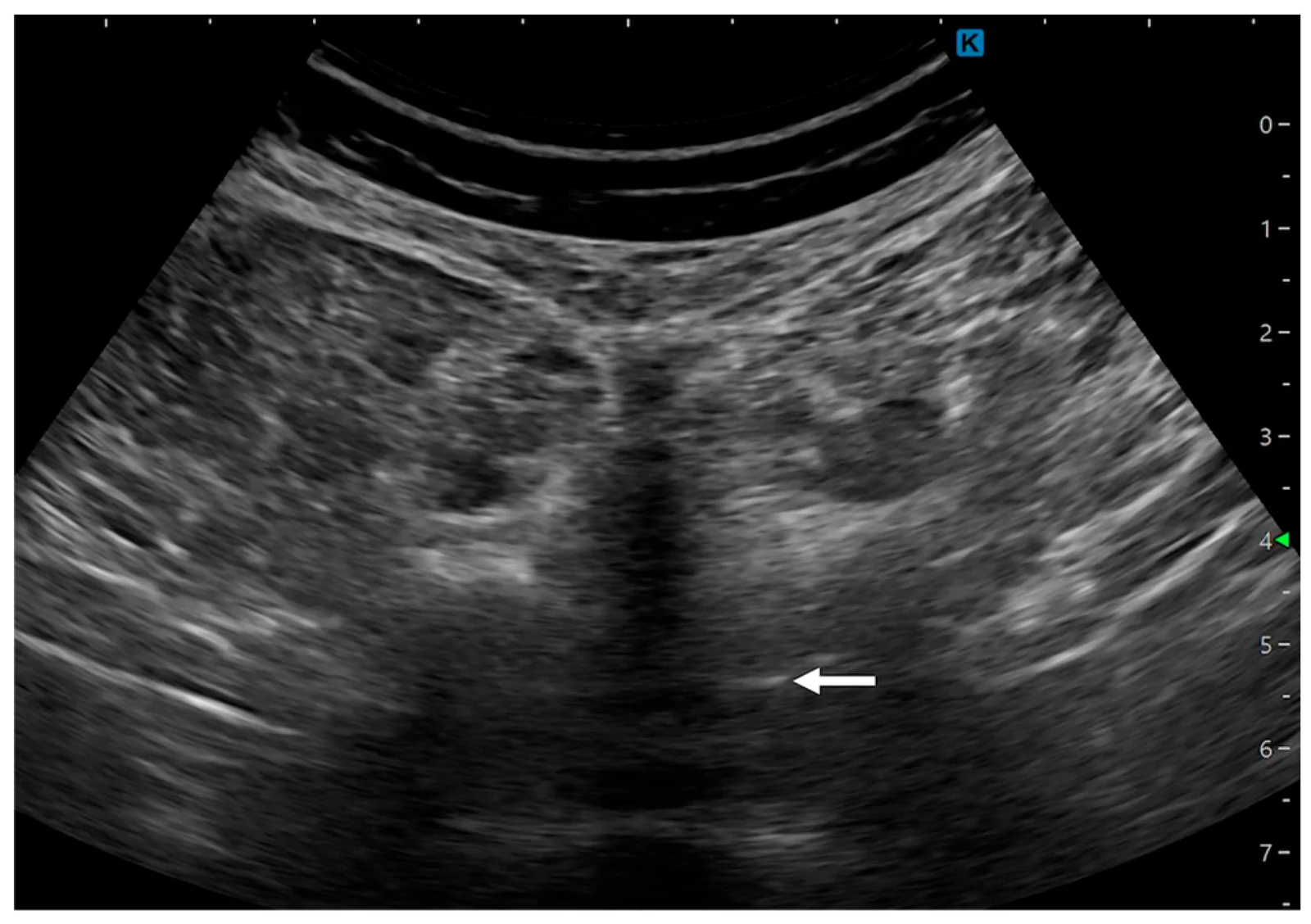
Lumbar ultrasonography.

On the day of the procedure, the L3/4 intervertebral space was identified with ultrasound, and the puncture depth was estimated at 54 mm after confirming the posterior complex (Figure 3). Spinal anesthesia was attempted at the L3/4 interspace using a 27-G pencil-point needle with the patient in the right lateral decubitus position. An anesthesia resident made two midline attempts, during which the needle contacted bone and could not be advanced; no paresthesia occurred. Before the third attempt, an experienced anesthesiologist reassessed the puncture site and needle trajectory using ultrasound. The third attempt was successful, with clear cerebrospinal fluid return and no paresthesia. We administered 2.4 mL of hyperbaric bupivacaine, 0.1 mg of morphine, and 10 μg of fentanyl. After intrathecal injection, a bilateral sensory block to the T4 dermatome was confirmed, and cesarean delivery was completed without conversion to general anesthesia. A continuous phenylephrine infusion was initiated at 1.0 mg/h after spinal anesthesia, gradually reduced after delivery, and discontinued 30 min after the start of surgery without subsequent hypotension. The neonatal Apgar scores were 6 and 8 at 1 and 5 min, respectively. The surgery was completed without complications (operative time: 67 min; anesthesia time: 94 min).

Thirty minutes after returning to the ward, the patient received 1000 mg of intravenous acetaminophen for postoperative pain, followed by 15 mg of intravenous pentazocine approximately 5 h later. Sensory and motor functions were assessed by the nursing staff until complete recovery from spinal anesthesia several hours after surgery, with no new neurological abnormalities. On postoperative day 1, she reported no neurological symptoms during the postoperative anesthetic round, and no neurological abnormalities were observed during the remainder of her hospitalization.

Lumbar computed tomography showing spina bifida occulta at L5 (arrow).

Preprocedural lumbar ultrasonographic image showing the L3/4 interspace and the posterior complex (arrow).

### Patient Perspective

The patient expressed gratitude for the anesthetic management and was satisfied that spinal anesthesia could be performed after detailed preoperative evaluation with MRI. She reported no neurological symptoms and was pleased with the overall perioperative experience.

## 3. Discussion

Spina bifida occulta (SBO) is a heterogeneous term encompassing conditions ranging from isolated non-fusion of the posterior vertebral arch to occult spinal dysraphism involving neural structures. Isolated posterior arch non-fusion is often an incidental radiographic finding without neurological deficits. Fidas et al. reported radiographic SBO in 23% of 2707 otherwise normal adults, illustrating that isolated posterior arch defects are relatively common and should not automatically be equated with clinically significant neural dysraphism [3]. In contrast, occult spinal dysraphism may include a low-lying conus medullaris, tethered cord, filum terminale abnormalities, spinal lipoma, dermal sinus tract, or split cord malformation [1,2]. From an anesthetic perspective, the principal concern is therefore not the vertebral defect itself, but the presence and location of associated neural abnormalities within the anticipated puncture range [1,2].

Before modern imaging became widely available, neuraxial anesthesia was often considered hazardous in patients with spina bifida because the position of the spinal cord and associated abnormalities could not be determined reliably. Neurological complications reported after neuraxial procedures were subsequently attributed in some cases to previously unrecognized abnormalities, including a low-lying conus medullaris, tethered cord, diastematomyelia, and spinal lipoma [1,2,4,5]. These observations contributed to the historical perception that neuraxial anesthesia should be avoided in patients labeled as having SBO.

Contemporary practice instead favors individualized assessment based on the underlying anatomy. MRI can determine the termination level of the conus medullaris and identify tethering, filum terminale abnormalities, spinal lipoma, and other intraspinal lesions [1,2]. Imaging findings may therefore either support neuraxial anesthesia by identifying an anatomically suitable puncture level or lead to its avoidance when neural structures are considered at risk [1,2]. Lumbar ultrasonography provides complementary procedural information by identifying intervertebral levels, assessing the posterior complex, estimating needle depth, and assisting selection of the puncture trajectory [2,6].

In the present patient, radiography and CT demonstrated an isolated posterior vertebral arch defect, whereas MRI confirmed a normally positioned conus medullaris terminating at L1 and showed no evidence of tethered cord, spinal lipoma, filum terminale abnormality, or other neural involvement. These findings distinguished an isolated bony abnormality from clinically relevant occult spinal dysraphism. Preprocedural lumbar ultrasonography was then used to identify the L3/4 interspace and assess the intended puncture site. Thus, MRI supported neurological risk assessment and anesthetic decision-making, whereas ultrasonography assisted procedural planning.

The approach used in this case does not represent a novel neuraxial technique. Crowe and Drew emphasized the importance of antepartum anesthetic assessment, review of relevant imaging, and individualized planning in parturients with structural spinal abnormalities [2]. The educational value of the present case lies in illustrating the application of these established principles to a parturient with an unrepaired isolated posterior arch defect who required scheduled cesarean delivery.

Previous reports are clinically heterogeneous with respect to the underlying spinal pathology, previous surgical repair, neuraxial technique, and availability of preprocedural imaging. Therefore, the present case should not be interpreted as evidence that spinal anesthesia is generally safe in all patients diagnosed with SBO. Rather, it illustrates how precise anatomical classification, complementary imaging, patient preference, and contingency planning may support individualized anesthetic decision-making in a selected patient.

## 4. Conclusions

In this patient with radiographic spina bifida occulta, MRI confirmed a normally positioned conus medullaris and excluded tethered cord and other neural abnormalities, while lumbar ultrasonography assisted identification of the intended puncture site. These complementary imaging modalities supported individualized anesthetic planning. The findings of this single case should not be generalized to all patients with spina bifida occulta.

## Data Availability

The data underlying this case report are not publicly available because they contain information that could compromise patient privacy. De-identified details may be available from the corresponding author upon reasonable request, subject to institutional approval.

## Abbreviations

The following abbreviations are used in this manuscript:

CT	Computed tomography
MRI	Magnetic resonance imaging
